# An iterative workflow for mining the human intestinal metaproteome

**DOI:** 10.1186/1471-2164-12-6

**Published:** 2011-01-05

**Authors:** Koos Rooijers, Carolin Kolmeder, Catherine Juste, Joël Doré, Mark de Been, Sjef Boeren, Pilar Galan, Christian Beauvallet, Willem M de Vos, Peter J Schaap

**Affiliations:** 1 Laboratory of Systems and Synthetic Biology, Wageningen University, Dreijenplein10, 6703 HB Wageningen, The Netherlands; 2 Department of Veterinary Biosciences, Division of Microbiology and Epidemiology, University of Helsinki, P.O. Box 66, FIN-00014 Helsinki, Finland; 3 INRA, UMR 1319, Micalis, 78350 Jouy-en-Josas, France; 4 Laboratory of Biochemistry, Wageningen University, Dreijenlaan 10, 6703 HB Wageningen, The Netherlands; 5 UMR U557 INSERM, U1125 INRA, CNAM, Université Paris 13, F-93017 Bobigny, France; 6 INRA, IsoCellExpress (ICE), UMR 1313 GABI, 78350 Jouy-en-Josas, France; 7 Laboratory of Microbiology, Wageningen University, Dreijenplein 10, 6703 HB Wageningen, the Netherlands

## Abstract

**Background:**

Peptide spectrum matching (PSM) is the standard method in shotgun proteomics data analysis. It relies on the availability of an accurate and complete sample proteome that is used to make interpretation of the spectra feasible. Although this procedure has proven to be effective in many proteomics studies, the approach has limitations when applied on complex samples of microbial communities, such as those found in the human intestinal tract. Metagenome studies have indicated that the human intestinal microbiome contains over 100 times more genes than the human genome and it has been estimated that this ecosystem contains over 5000 bacterial species. The genomes of the vast majority of these species have not yet been sequenced and hence their proteomes remain unknown. To enable data analysis of shotgun proteomics data using PSM, and circumvent the lack of a defined matched metaproteome, an iterative workflow was developed that is based on a synthetic metaproteome and the developing metagenomic databases that are both representative for but not necessarily originating from the sample of interest.

**Results:**

Two human fecal samples for which metagenomic data had been collected, were analyzed for their metaproteome using liquid chromatography-mass spectrometry and used to benchmark the developed iterative workflow to other methods. The results show that the developed method is able to detect over 3,000 peptides per fecal sample from the spectral data by circumventing the lack of a defined proteome without naive translation of matched metagenomes and cross-species peptide identification.

**Conclusions:**

The developed iterative workflow achieved an approximate two-fold increase in the amount of identified spectra at a false discovery rate of 1% and can be applied in metaproteomic studies of the human intestinal tract or other complex ecosystems.

## Background

The human intestinal tract is colonized since birth by a large number of microbes, together making a complex ecosystem, even considered an organ by itself [1]. Many studies indicate a pivotal role for the intestinal microbes in carbohydrate metabolism, production of vitamins, inflammatory response regulation, fat metabolism and other biological processes of the human host [2,3]. In adults, the community consists of around 10^14 ^cells [4-6], with a complexity that is predicted to include over 5000 microbial species [3]. While recent progress has been made in characterizing the genomes of around 200 intestinal species in the Human Microbiome Project (HMP) [7], the vast majority has not yet been cultured. Hence, these are known as phylotypes as their presence can be deduced from molecular markers such as 16S rRNA and other nucleotide sequences. This approach has shown that most of the intestinal phylotypes belong to a limited set of phyla, including the Firmicutes, Bacteroidetes, Actinobacteria, Proteobacteria and Verrucomicrobia [5]. In healthy adults the intestinal microbiota fluctuates around a stable individual core of phylotypes that are affected by host genetics, environmental and stochastic factors [3]. High throughput metagenomics studies, such as that of the recently reported MetaHIT project, have indicated that the human gut microbiome contains a gene repository that is estimated to be over 100 times the number of genes encoded by the human genome [8].

The current developments in high-throughput *omics *techniques allow for unique insight in the functions of this community that can be predicted via metagenomics and established at the biochemical level. Proteomics of isolated microbes is an established and powerful method to determine global expression profiles [9]. However, *meta*proteomics i.e studying the proteome of a complex environmental system like the human intestinal microbiota is an emerging field within the area of proteomics that is characterized by a high level of complexity [10,11]. Common high-throughput spectral interpretation algorithms use peptide spectrum matching to link the raw data obtained from a mass spectrometer to large listings of peptides that are possibly represented in the data. Generation of theoretical spectra from these peptides present in the database and matching them with the obtained data forms the basis of these algorithms [12]. Thus, in order to properly identify the spectra, one needs to know very accurately which peptides one can expect. The complex gut microbial proteome is, however, far from defined, as the HMP is still advancing [7,8]. Moreover, the distribution of species is dynamic and it differs between individuals [3,13]. A common method to circumvent the lack of a defined proteome is to use proteomes from closely related species in so-called *cross-species protein identification*. This is reported to lead to relatively high false discovery rates however, and should be considered a sub-optimal solution [14]. Another approach is to exploit the metagenomics developments but this approach suffers from the limited coverage of the present databases. An ideal case is the use of available sequence data of the sample that is to be analyzed by metaproteomics. This matched metagenome could serve as a basis for the predicted metaproteome by naive six-frame translation and basic filtering of the obtained hypothetical proteins for the presence of trypsinated peptides of sufficient size. Since most of the predictions will be incorrect as it fills the search space with noise, this approach may also either suffer from a high false discovery rate or results in a low proteomics identification efficiency.

Since both approaches suffer from unacceptable high-false positive rates, which lead to low accuracy in the identification of proteins, we aimed to develop an iterative workflow based on a synthetic metagenome from over 200 intestinal species that would increase the sensitivity of peptide identification by peptide spectrum matching of the metaproteomics data. Synthetic metagenomes have already been successfully applied in the analysis of less complex microbial proteomes, [15] and biofilms, reviewed in [16] and recently in the analysis of biostimulated microbial communities from field experiments [17]. For the complex microbial proteome of fecal samples VerBerkmoes et al., [18] successfully used a mixture of gut microbial metagenome sequence data supplemented with a synthetic metagenome constructed from known gut inhabitants representatives. The human gut microbial gene catalogue [8] is however increasing exponentially and thus there is need for alternative approaches that keep the false discovery rate within limits while still maintaining high proteomics identification efficiency.

In this study a synthetic metagenome setup is used as a starting point in an iterative approach. This approach combines the power of sequence diversity contained in metagenomic diversity while keeping the reliability of predicted proteins high and hence the search space small, which limits false discovery rates. To provide proof of concept for this approach, we determined the metaproteomes of two fecal samples from healthy adults that had also been characterized by metagenomic sequence analysis.

## Results and Discussion

To develop the iterative workflow for identifying mass spectrometric data from the intestinal metaproteome, we first assembled a synthetic metagenome. This was done by selecting a set of genomes from 216 known gut inhabitants that were available, fully sequenced, and annotated [19] (for a listing see additional file 1). Subsequently, we collected a metaproteomics dataset from fecal samples from two French healthy subjects, termed NO1 and NO3, which had been analyzed by metagenome sequencing. Finally, we benchmarked the developed iterative workflow by comparing the predictions obtained with other search strategies.

### Phylogenetic Analysis of Two Human Metagenomic Datasets

**Figure 1:**
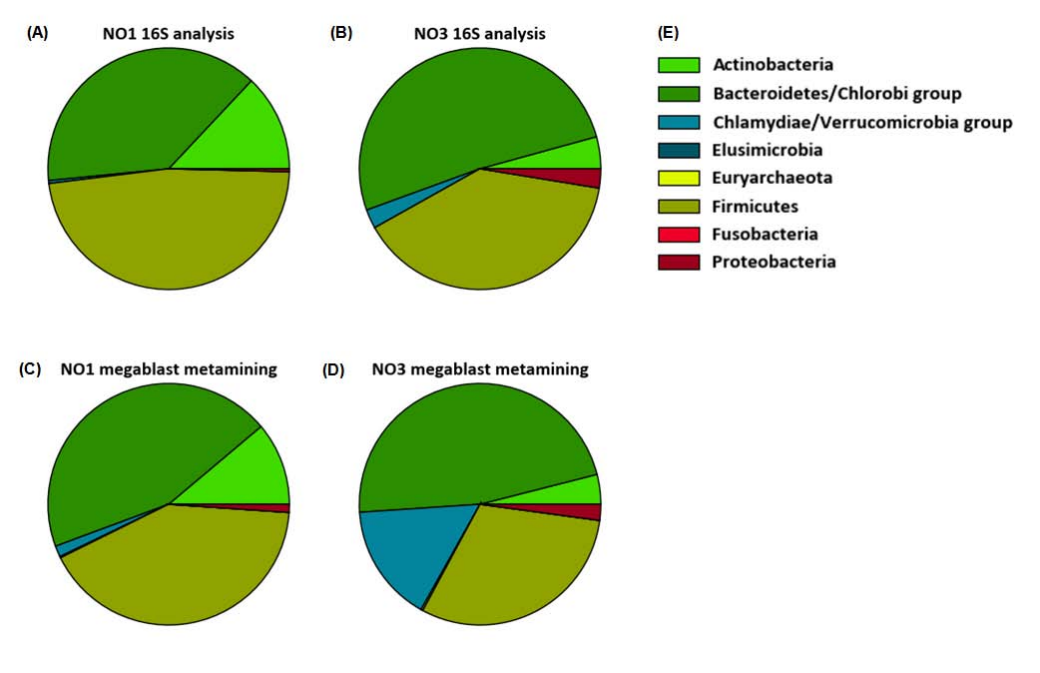
**16S rDNA and Meta-mining analysis of matched metagenomes NO1 and NO3**. A and B: 16S rDNA abundance analysis of the two metagenomes. The analysis shows the presence of Bacteroidetes, Firmicutes and Actinobacteria as dominant phylotypes in the gut samples. C and D: Meta-mining of the traces, using a set of 216 annotated high quality genomes from known gut inhabitants using a sequence similarity threshold of >90% over >300 nucleotides. 46.2% of the NO1 traces and 41.8% of the NO3 traces were matched in this approach. The analysis confirms the distribution over the phyla measured using 16S rDNA analysis. E: Color code key

The two matched metagenomes from the subjects NO1 and NO3 were determined by high fidelity Sanger sequence analysis and contained 128,441 and 120,415 sequence trace files respectively, giving in total 169,762,767 and 162,162,286 nucleotides per metagenome. The phylogenetic diversity of these samples was determined by 16S rRNA sequence analysis of the metagenomic data sets (Figure 1A and 1B). This was compared to an abundance analysis of the metagenomic sequences using the synthetic metagenome as reference set (Figure 1C and 1D, see Materials and Methods section for details). The results indicate the presence of Bacteroidetes, Firmicutes, Actinobacteria, Verrucomicrobia and Proteobacteria as the dominant phylotypes in these gut samples (Figure 1). Moreover, the high congruency between the different approaches testifies for the representativeness of the synthetic metagenome used in this study.

Both phylogenetic analysis methods (Figure 1) revealed significant difference between the two samples in abundance of Actinobacteria, Proteobacteria and Verrucomicrobia and both methods suggest that the Verrucomicrobia are more present in the NO3 sample. Remarkably, based on the abundance analysis of the metagenomic sequence trace files the Verrucomicrobia were highly prominent in the NO3 sample (representing approximately 12% of the total community). The metagenomic sequence trace files from this group all seem to originate from *Akkermansia muciniphila-like *species. *A. muciniphila *is a common bacterial component of the human intestinal tract and is reported to reach densities up to approximately 3% in the human colon [20]. It is unlikely that the discrepancy between the direct diversity analysis and the metagenome mining results derived from inappropriate annotation of the metagenome information, as *A. muciniphila *belongs to the Verrucomicrobia, which is a deeply rooted phylum, and changing the cut-off values for the average nucleotide identity from 90 to 95% still predicted 6% abundance of *A. muciniphila*-like bacteria (data not shown). Hence we assumed that this discrepancy is a result of a cloning bias of the 16S rRNA versus genomic fragments of the *A. muciniphila*-like bacteria. Such cloning biases have recently been reported in a model study comparing different metagenomic sequencing approaches [21].

### Metaproteomics Data Collection and its Analysis Using Naive Translations of the Matched Metagenomes

**Figure 2:**
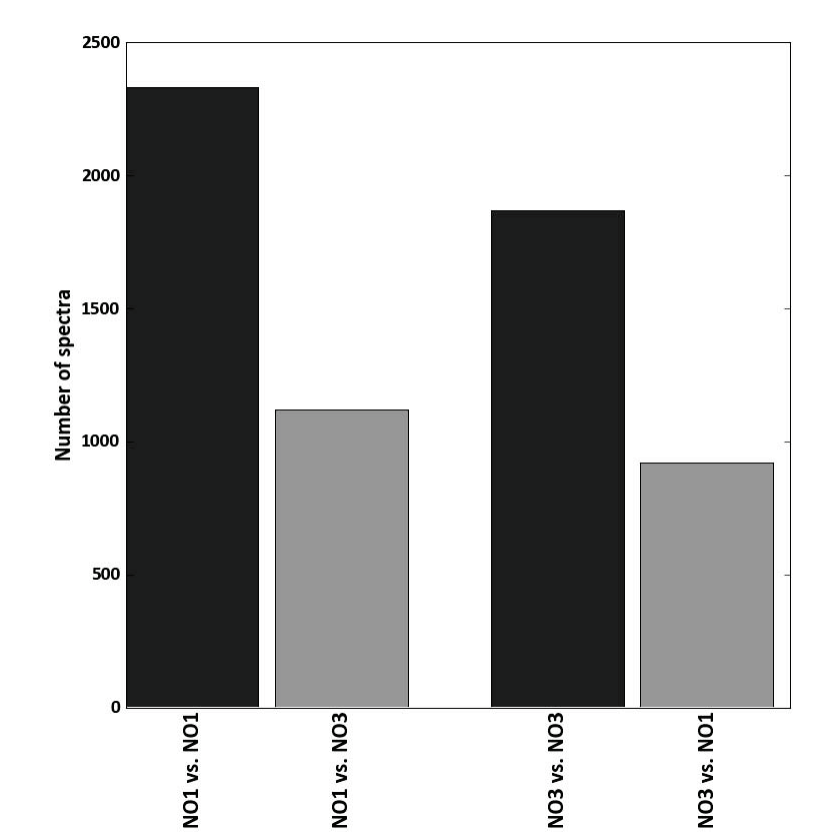
**The effect of using matched metagenome in peptide spectrum matching**. Total number of Peptide Spectrum Matches (PSM) with matched microbial genome sequences obtained from stool samples of human volunteers NO1 and NO3. The spectra from the two samples were identified using a naive translation of their respective matched metagenomes (black bars) and cross-validated with the naive translation of the non-matching metagenome (grey bars).

Proteins extracted from the microbial fractions isolated from the fecal samples of subjects NO1 and NO3 were separated by 1-D SDS-PAGE followed by liquid chromatography-mass spectrometry (LC-MS/MS) analysis of trypsin-treated gel fractions. This resulted in the collection of a set of 41845 and 45042 MS/MS spectra in the NO1 and NO3 samples respectively. Six-frame naive translations were made from the matched NO1 and NO3 metagenomes, filtering out small peptides. Peptide Spectral Matching (PSM) was performed on the MS/MS data using the NO1 and NO3 naively translated metagenomes as single search spaces. False positives were determined using reversed versions of each database [22] and the results of all PSM analyses are reported at a fixed peptide False Discovery rate (FDR) of 1%. The peptide search against the matched metagenomes resulted in 2,331 peptide hits for NO1 and 1,870 peptide hits for NO3 (Figure 2). Moreover, the search against the un-matched metagenomes resulted in only 1,120 and 922 peptide hits for the NO1 and NO3 metaproteomes, respectively (Figure 2). Remarkably, the use of the matched metagenome resulted in a more than two fold increased number of identified MS/MS spectra compared to the un-matched metagenome, demonstrating the importance of using a search database where the listed sequences precisely match the peptides present in the sample. However, the number of peptides present might be even under-estimated when using the naive translations as search space as these have the drawback that the pseudogenes vastly outnumber true genes. This results in a large number of false positives that only can be eliminated by increasing the threshold and hence limiting the discovery success. Therefore, an alternative approach was developed based on an iterative analysis of the metaproteome data using a synthetic metagenome database as described below.

### Development of an Iterated Search Method Using a Synthetic Metagenomic Data Set

As indicated above, a representative synthetic metagenome data set was constructed and used for developing an improved method for metaproteomics data analysis.

**Figure 3:**
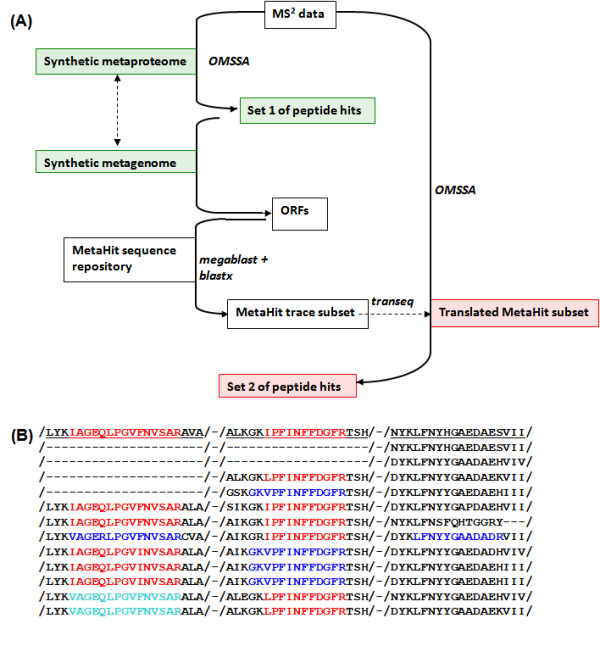
**The bioinformatics workflow of the proposed method**. A: Peptide spectral matching with the 'synthetic metaproteome' leads to peptide hits. The ORFs underlying the hitting peptide sequences are used to select a subset of the metagenomic pool of sequence data (in this case MetaHIT sequence repository). The two-step selection of megablast and blastx reduces the computational load and allows the metagenomic pool to be large. The selected subset is naively translated and is used as a search space for a next round of peptide spectral matching, generating set 2 of peptide hits. Note that many elements of the workflow can be altered to fit other metaproteomics studies. B: Alignment of naive translations of selected traces with a synthetic metagenome starting sequence assigned to COG074. Underlined: domains of the synthetic metagenome starting sequence; Red: peptides spectrum matches using the synthetic metagenome peptide database; Blue: variant peptides detected in selected naively translated MetaHit sequence trace files. Leucine (L) and isoleucine (I) are isobaric and can therefore not be detected separately.

The sequence variability present in the predicted synthetic proteome cannot be fully covered, as the species diversity of the intestinal microbiota is enormous. Moreover, in the analysis of shotgun proteomics data with PSM algorithms, an exact match between the experimental data and a theoretical spectrum from a theoretical peptide from the database is necessary. Peptides containing a single amino acid polymorphism are not detected if they are not contained in the database. To circumvent this limitation, it is necessary to cover the sequence variability of the proteins in the sample. With the release of sequence repositories from the high throughput metagenomic studies, such as the MetaHIT project, an enormous amount of gut metagenome data is available [8]. These repositories are increasing in size and hence difficult to use directly in a naive translation approach as their complexity is increasing exponentially. Furthermore, faithful annotation of the data is hampered by the inability to efficiently transform sequence data completely into contigs of sufficient length that capture complete protein encoding genes [8,23]. Moreover, there is no way to deal with the inevitable occurring sequencing errors associated with the new generation technology sequencing approaches [24]. Furthermore, standard methods of FDR calculation using decoy databases derived from search databases of this size are increasingly impractical, notably as the computing time is limiting. The iterative workflow we propose here is based on a combination of the synthetic metagenome described above and a metagenome repository without any form of assembly, annotation or a priori translation (see Figure 3). We selected the MetaHIT dataset as a basis as this is by far the most comprehensive analysis to date [8]. It is evident that the synthetic metagenome selected here may be increased in size as the HMP is continuously progressing.

PSM analysis of the MS/MS data is performed in a first step using the synthetic metaproteome. Since these genomes are reliably annotated, we are able to use the DNA gene-sequences coding for the peptides identified in the first PSM run to retrieve homologous sequence trace files from the MetaHIT repository. To reduce the computational load this enrichment procedure is done in two steps (Figure 3). The first step using discontiguous megablast with coding spaced seeds [25] performs a first selection of these sequence trace files at DNA level. The second step using blastx performs a comparison of this selection on amino-acid level, thus ensuring homology between the protein sequence from the synthetic metagenome selected by the initial peptide spectrum match and the MetaHIT sequence trace file on peptide level. Next the selected sequence trace file is translated and recorded in a new peptide database. These new peptide databases are created for each individual MS data sample. The databases are then used for a next round of PSM analysis.

**Figure 4:**
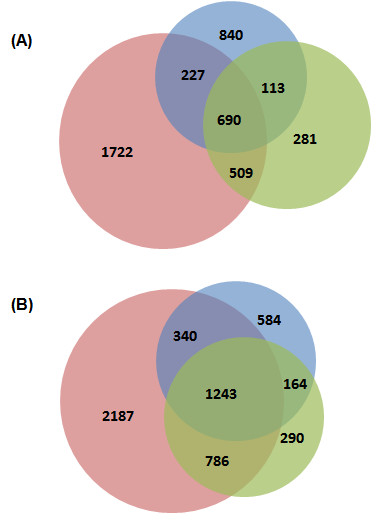
**Results from three different approaches to peptide spectrum matching of shotgun meta proteomics data**. Spectra from NO1 (A) and NO3 (B) were identified in separate OMSSA runs using the 'synthetic metaproteome' (green), naive translation of the matched metagenome (blue) and a translated metagenomic subset (red) as search space. Combining the search results from the synthetic metagenome search and the results from the search against the translated metagenomic subset, the proposed method (Figure 3) yields the highest spectrum identification rate. As expected, most of the spectra identified using the 'synthetic metaproteome' are also identified using the translated subset.

Application of this iterative search workflow showed an increase in spectral identifications when compared to that obtained with the synthetic metagenome and the earlier described matched metagenome searches. The iterative method resulted in 5,010 and 3,542 identified peptides in the NO1 and NO3 samples, respectively. Compared to each of the two other methods, this represents an approximately 2-fold increase (Figure 4). An important factor contributing to the increased number of identifications is the increase in sensitivity of the iterative method compared to a direct synthetic metaproteome approach. The sequence trace files enable the PSM algorithms to search the data using a broad sequence variability. (Figure 3). Another factor is the increase in specificity of the enrichment procedure with respect to a naive translation approach. As the number of incorrectly predicted proteins is drastically reduced by the method of sequence trace file selection before they are translated, the FDR is also drastically reduced.

The Open Mass Spectrometry Search Algorithm (OMSSA) search against the matched metagenomes resulted in 840 and 584 identified peptides for NO1 and NO3 respectively which could not be identified by the iterative search approach. These hits are peptides, which do not have an apparent homolog in the synthetic metagenome. However, this loss of information can be taken into account as in a standard experiment a matched metagenome is not available.

The enrichment procedure also minimizes the impact of an initial false discovery. Sequence trace files selected by a true peptide match will in turn produce additional PSMs (Figure 3) and thus increase the relative weight of the presence of particular molecular function in a matched gut metaproteome. Trace sequence files selected by a random hit generally will not produce additional PSMs for the same molecular function. These concepts together contribute to the accuracy of the method and the increased number of identified spectra. Moreover, as the size of protein sequence databases is constantly growing, the success of the identifications is expected to increase accordingly using the described iterative method that accommodates the dynamics in database development.

### Molecular Function Prediction of the Metaproteomics Data

**Figure 5:**
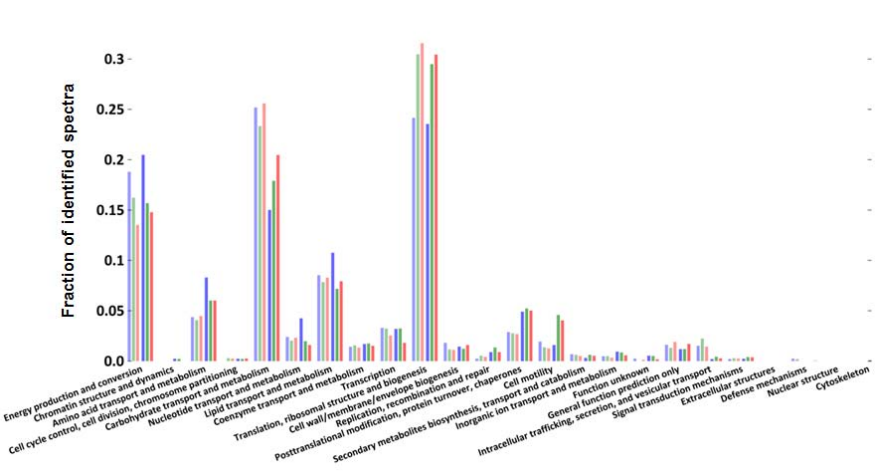
**The distribution of the spectra over the COG categories**. Left three bars represent spectra counts per COG category for the NO1 sample right three bars show spectra counts per COG category for NO3 sample. Blue: Peptide database is derived from direct naïve translation of traces; green: Peptide database is derived from the non-redundant synthetic metagenome; red: Iterative Peptide database

To illustrate the power of the described metaproteomic approach in combination with the iterative search method, we predicted the functions of the intestinal microbiota isolated from the subjects NO1 and NO3. For this purpose we performed a blast search of the trace hit sequences against the COG Clusters of Orthologous Proteins of Comparable Molecular Function [26] database (Figure 5 and additional file 2) and assigned a COG to each identified spectrum. Molecular function abundance estimates were determined by summing the COGs over all spectral counts and grouping COGs into COG families (see Materials and Methods section for details). The iterative search method resulted in a similar distribution over the COG families as that obtained with the synthetic metaproteome-search method, reflecting the approach taken (Figure 5). Remarkably, the distribution over the COG families between the NO1 and NO3 samples is highly similar. The COG categories *Translation*, *Energy production and conversion *and *Carbohydrate transport and metabolism *count for over 50% of the spectra in each sample. Furthermore, COG categories *Amino acid transport and metabolism*, *Nucleotide transport and metabolism *and *Lipid transport and metabolism *are abundantly present. A similar distribution of COG families based on metaproteomes was recently reported for a Swedish adult twin pair [18]. It reassures the role of the gut microbiota in carbohydrate metabolism and harvesting and conversion of other nutrients. Moreover, the elucidation of the full range of microbial functions allows studies to gain insight in the relation between gut microbial community and obesity or other inflammatory disorders.

### Metaproteomic Mining Points to a Specific Role of *A. muciniphila*-like Bacteria in the Intestinal Tract

**Figure 6:**
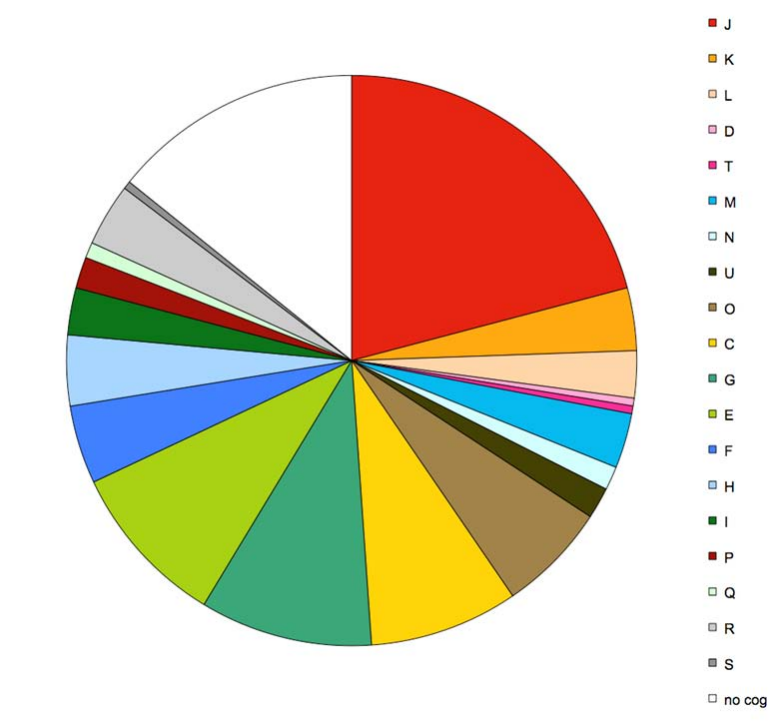
**COG functional category distribution of *A. muciniphila*-like bacteria in subject NO3**. Apart from the obvious housekeeping functions, the largest COG groups include proteins predicted to be involved in carbohydrate transport and metabolism (G) as well as amino transport and metabolism (E).

The identified metaproteome can also be used to focus on the proteome of phylogenetically deeply rooted microbial taxa as these stand out in the blast analyses. An example is *A. muciniphila*-like bacteria that is a single intestinal representantative of the deeply rooted Verrucomicrobia and has recently been characterized at the genome level (van Passel et al. submitted). The traces from the MetaHIT pool selected by a PSM were phylogenetically analyzed by a meta-mining approach for the relative presence of proteins derived from *A. municiphilia-*like bacteria. This resulted in the detection of a total of 9 such proteins in the NO1 metaproteome while close to 200 of such proteins were found in the NO3 metaproteome (see additional file 2). This reflects accurately the observed abundance of *A. muciniphila*-like bacteria in the different metagenome datasets (see Figure 1). Close inspection of the large dataset from the NO3 subject shows a specific COG distribution of the peptides derived from *A. muciniphila*-like bacteria (Figure 6). Apart from the obvious housekeeping functions, the largest COG groups included proteins predicted to be involved in carbohydrate transport and metabolism as well as amino acid transport and metabolism. This is compatible with the observation that *A. muciniphila *is capable of using mucin as carbon and nitrogen source [20].

Moreover, as these proteins include various mucinases, it testifies for predicted activity and function of *A. muciniphila*-like bacteria in the intestinal tract.

## Conclusions

The wealth of information contained in the intestinal metaproteomes becomes increasingly accessible as technical and analytical methods for its analysis mature. Although there are still limitations in peptide identification, we describe here a novel approach that increases the accuracy of peptide identification from shotgun proteomics data significantly. The setup of the iterative workflow is highly dynamic, which allows capitalizing on the developing databases. Moreover, it is sufficiently general to make it applicable to study metaproteomes of other ecosystems. The developed iterative workflow was benchmarked to other approaches using data from two human intestinal metaproteomes, showing superior identifcation power and its applicability in defining functions of intestinal bacteria.

## Materials and methods

### Study Material

Fecal material was obtained from subjects NO1 and NO3 from the Micro-Obes - Human Intestinal Tract Metagenomes. NO1 and NO3 were two healthy lean male volunteers aged 63 and 61 respectively, without symptoms of gastrointestinal disease, family history of gastrointestinal disease, or medication use. They were recruited from the SU.VI.MAX2 cohort, in which they had been followed over 8 years for their healthy nutritional and lifestyle habits [27]. The SU.VI.MAX 2 study was conducted according to the guidelines laid down in the Declaration of Helsinki and was approved by the Ethical Committee for Studies with Human Subjects of Paris-Cochin Hospital (CCPPRB n° 2364) and the Comité National Informatique et Liberté (CNIL n° 907094). Written informed consent was obtained from all subjects.

### Liquid chromatography tandem mass spectrometric analysis

Bacteria were separated from fresh fecal material from subjects NO1 and NO3 by flotation in a preformed Nycodenz continuous gradient adapted from [28] and stored at -80°C. Proteins were extracted from bacterial pellets by chemical lysis. Freshly prepared buffer containing 8.75 M urea (Pharmacia), 2.5 M thiourea (Sigma), 5% (w/v) CHAPS (Sigma), 75 mM DTT (Sigma) and 31.25 mM dihydrate spermine base (Fluka) was added to each frozen bacterial pellet. The pellets were dispersed by vigorous vortexing and incubated at room temperature for 1 h with periodic vortexing. The lysates were centrifuged at 45,000 rpm for 1 h at 18°C. Protein concentration of the supernatant was determined using the GE Healthcare 2-D Quant Kit. After neutralizing with concentrated HCl, the protein solutions were stored at -80°C.

Protein solutions were buffer exchanged and per sample 50 μg of proteins were fractionated on a 4-12% gradient gel and lanes fractionated into 20 pieces. Proteins were reduced with 50 mM (DTT) in 50 mM ammonium bicarbonate (NH_4_HCO_3_) for 1 h at 60°C, alkylated with 100 mM iodoacetamide (IAA) in 50 mM NH_4_HCO_3 _for 1 h at room temperature and digested with 0.2 μg trypsin per gel piece at room temperature over night. Peptide solutions were acidified with trifluoroacetic acid to pH 2 and centrifuged. 18 μl of supernatant were loaded on a 0.1×30 mm reversed phase column and peptides were eluted to an 0.1×300 mm analytical reversed phase column with an acetonitrile gradient from 9 to 34% and a fixed concentration of formic acid in 50 minutes (Proxeon nLC). The eluent was subjected to an electrospray potential via a coupled platinum electrode. MS spectra were measured on an Orbitrap (Thermo Electron, San Jose, CA, USA) and MSMS scans of four most abundant peaks were recorded in data-dependent mode in coupled LTQ.

### Bioinformatics

#### Synthetic metagenome/metaproteome assembly

The synthetic metaproteome and synthetic metagenome were assembled by taking the known gut inhabitants described in [19] and the gut microbiota listed on the NCBI FTP server (ftp://ftp.ncbi.nih.gov/genomes/Bacteria/) for which both genomic information and annotated proteome information was available and (nearly) complete. Selected microbial proteomes are listed in additional file 1.

#### Synthetic metaproteome redundancy minimization

All protein sequence databases have been made non-redundant (in the case of the synthetic proteome at the level of subspecies, e.g., when *Lactobacillus reuteri *MM4-1 and *Lactobacillus reuteri *SD2112 share a protein sequence exactly, the sequence is only in the database once, however redundancy with other *Lactobacilli *is ignored. This eliminated most of the redundancy in the synthetic metaproteome), leading to lower (better) e-values in PSM runs and shorter computational times.

#### Abundance analysis of the metagenomic sequences

Metagenomic sequence data was compared to the synthetic metagenome using 'megablast' [29], with default settings. Reported hits are above a 95% average nucleotide identity threshold. The synthetic metagenome allowed extraction of species to determine lineage of the metagenomic data (Figure 1).

#### Metagenomic data

The pool of metagenomic traces consisted of the MetaHIT traces found at ftp://ftp.ncbi.nih.gov/pub/TraceDB/human_gut_metagenome/ (latest archive 13th of November 2009, in total 3,148,461,906 basepairs in 2,415,707 traces).

#### Naive translations of trace sequence data (i.e. of subset of MetaHIT and NO 1 (NCBI genome project 33305) & NO3 (NCBI genome project 33307) matched metagenomes)

Naive translation was done in six-frames, and naive translations shorter than 25 amino acids or having no fully tryptic peptide of length > 5 amino acids were filtered out (Python regular expression "[KR](?!P)([^K^R]|([KR](? = P))){5,}[KR](?!P)").

#### Decoy databases

Decoy databases for FDR estimation were created by reversing each individual protein sequence according to [22] and tagging the deflines to aid further analysis.

#### Peptide spectral matching

Peptide spectral matching was performed for the MSMS spectra of each gel piece using stand-alone version 2.1.7 of OMSSA [30] with fixed modification "carbamidomethyl C" (option "-mf 3"), variable modifications "oxidation of M" and "carboxymethyl C" (option "-mv 1,2"), product mass tolerance 0.4 Da (option "-to"), precursor mass tolerance 0.03 (option "-te") and maximum allowed E-value in hit list of 0.1 (option "-he").

#### FDR estimation

FDR estimation was done per sample/search database combination according to [22]. FDR estimation for which too few hits were generated used the score of the best False Positive as threshold with upper bound OMSSA e-value of 0.01 for subsequent data analysis.

#### Iterated search method

Iterated searching was done by (i) taking all PSM hits for all respective MSMS data sets searched against the synthetic metagenome search with e-value < 0.01 (excluding reversed hits from the decoy peptide database), (ii) megablasting the genomic sequences from those hits against the metagenomic pool with option "coding spaced seeds" of length 12 (option "-N 0 -W 12 -t 21") for optimal results [25,31] and maximum reported e-value 0.01 (option "-e"). Megablast output was parsed and only sequence trace files giving 50% identity over 150 nucleotides or better were retained for the next filtering round. (iii) The retained trace files were compared using blastx against the protein sequences from the PSM hits in (i). Output was parsed and only trace files having 80% identity over 50 amino acids or better with a subject sequence were used. (iv) The sequence trace files remaining were three-frame translated. The correct strand could be inferred from the blastx search and three-frame translation mitigates the problem of frame shifts in low-quality sequencing data. Translation was followed by basic ORF filtering described earlier at "naive translations". The translations were combined into a new search database used for the second PSM round again using an e-value < 0.01.

#### COG assignment

Sequences were searched using blastp against the COG database (NCBI, ftp://ftp.ncbi.nih.gov/pub/COG/COG/) and assigned the COG of the best hit if the hit had an e-value better than 10^-10^.

#### Computational details

All processing was done by in-house python (version 2.6) software except where usage of other software is stated. Data warehousing was done using MySQL (version 5.0.75). Machines used were an AMD Sempron 3000+ (1.8 GHz, 1 GB) for development and an Intel Xeon E5320 (8× 1.86 GHz, 8 GB) as deploy server, both running Linux 2.6 kernels.

## Authors' contributions

PG recruited the subjects. CK, JD, CJ, and WMdeV planned and PD, CK and SB performed the study. KR and PJS developed and performed the bioinformatics analysis. MdeB helped with the biological interpretation of the data. KR, PJS, CK and WMdeV wrote the paper with contributions from all authors. All authors read and approved the final manuscript.

## Supplementary Material

Additional file 1 **List of selected microbes used for construction of the synthetic metagenome**. Click here for file

Additional file 2 **COG annotation of matching mass spectra found**. File is in comma-separated value Click here for file
